# Physical Access Related User-Friendliness of Sexual and Reproductive Health Services for Women With Disabilities in Various Hospitals in a City in North India: An Integrated Qualitative and Quantitative Study

**DOI:** 10.7759/cureus.34276

**Published:** 2023-01-27

**Authors:** Ruchi Sharma, Ajit Kumar, Vanita Suri, Sukhpal Kaur, Amarjeet Singh

**Affiliations:** 1 Community Medicine and School of Public Health, Postgraduate Institute of Medical Education and Research (PGIMER), Chandigarh, IND; 2 Physiotherapy, Postgraduate Institute of Medical Education and Research (PGIMER), Chandigarh, IND; 3 Obstetrics and Gynecology, Postgraduate Institute of Medical Education and Research (PGIMER), Chandigarh, IND; 4 Nursing, National Institute of Nursing Education, Postgraduate Institute of Medical Education and Research (PGIMER), Chandigarh, IND; 5 Department of Community Medicine, Shri Ram Murti Smarak Institute of Medical Sciences (SRMSIMS), Bareilly, IND

**Keywords:** hospital care, women's health, user friendliness of obstetrics & gynecology services, women with disability, disabled friendliness, disability, access survey and audit

## Abstract

Introduction: Access to optimum health care services is vital for every woman. Women with disabilities (WWD), in particular, face multiple discrimination and social exclusion on this issue. The objective of the study was to evaluate the user-friendliness of sexual and reproductive health (SRH) services in terms of the physical access to health facilities in various hospitals in a city in north India.

Methods: This cross-sectional evaluation study was conducted during 2013-2017 in five purposively selected major government hospitals in a city in north India. A disabled friendliness evaluation tool was used to analyze the barriers to physical access in terms of approach to SRH care facilities. Data analysis was done through Microsoft Excel (Microsoft, Redmond, WA, USA) and content analysis.

Results: Overall, 270 respondents aged 15 years and above with at least 40% disability were interviewed for the study. Lack of access to SRH care in hospitals (infrastructure/equipment/communication/transport) was the main barrier (46.36%) reported by WWD. Most of the hospitals lacked any special provisions for people with disability (PWD). Proper ramps, stairs, toilets, etc. were not present in hospitals as denoted by their verbatim responses.

Conclusions: All the hospitals scored low on access to sexual and reproductive health services for WWD.

## Introduction

Even in the 21st century, the healthcare needs of women are not adequately met. All these issues are much more pronounced for women with disabilities (WWD). A freely accessible healthcare system without obstacles is a prerequisite to mainstream WWD. Globally, people with disabilities (PWD) face significant problems in their routine lives. PWD have an equal right to sexual and reproductive health (SRH) needs and desires. But, this aspect has been largely disregarded by our society. WWD face barriers to SRH care everywhere, which constrains their involvement in society-level activities. Barriers to health care faced by WWD are not necessarily a part of having a disability but reflect the lack of social support, legal protection, understanding, and empathy. These barriers include political, economic, structural, and socio-cultural factors. Other barriers are the cost of treatment, lack of appropriate services and equipment, and physical access to health facilities [1]. 

Recently, plenty of discussion about disability-related issues has taken place globally. As most of the focus of such a forum remains on disability, it overshadows the SRH rights of PWD. Globally, at least one billion PWD face significant problems in their routine lives [1]. India ratified the United Nations Convention on the rights of PWD, yet little was done to protect their rights.

Reproductive healthcare services can be improved to accommodate WWD easily if healthcare providers and health planners work together. It will help in mainstreaming WWD within society. There is little evidence regarding the degree of access of WWD in India to SRH services. There is also a lack of research that asks them about their experiences and opinions about the SRH services they receive [2]. Because most health services in hospitals still lack a disabled-friendly attitude, design, and facilities, a serious effort to address the matter is needed to ensure the welfare of WWD. It will enable planners to suggest measures to remove them. We can find ways to make it easier for WWD to get into the hospitals, to use the equipment, increase awareness about SRH needs of WWD and improve the attitudes of health workers/family members toward WWD. Most of these changes are not difficult or expensive. Since no such research on this topic has been done in India, we aimed to explore the domain related to the problem that WWD face in accessing SRH services. 

## Materials and methods

This cross-sectional evaluation study was conducted during 2014-2017 in all major government hospitals (n=5) in a city in north India. A disabled friendliness evaluation tool was used to analyze the barriers to physical access in terms of approach to health care facilities. A pre-tested study tool (Access survey and audit checklist), designed by the Rehabilitation Council of India (RCI) and Samarthyam-National Centre for Accessible Environments was used [3]. This checklist was modified and scored according to our study environment. Study domains included evaluation of hospital services for accountability and outdoor and indoor environments. The scoring criteria were validated by circulating the tool among PWD, experts in the field of public health/obstetrics and gynecology (OBG)/physical rehabilitation medicine, representatives from self-help groups, architects, and civil engineers. This tool was used for the study after pre-testing. During the evaluation, special focus was given to departments. Facilities were given grades according to their obtained score. The maximum attainable score was 280 (Table 1).

**Table 1:** Grading of accessibility score of obstetrics and gynecology (OBG) services in various hospitals

Grade	Score
0-70	Poor
71-140	Average
141-210	Good
210-280	Excellent

Quantitative data analysis was done through Microsoft Excel (Microsoft, Redmond, WA, USA). Besides this, a qualitative approach through in-depth interviews (IDI) was also adopted to understand the processes associated with accessing SRH services and the challenges encountered in accessing these services from the point of view of WWD themselves and their caregivers. For this purpose, the investigator, a public health professional, purposively selected WWD who were suffering from SRH problems as revealed by them (clinically diagnosed/self-reported) in OBG clinics of the study hospitals.

A total of 270 WWD respondents, aged 15 years and above with at least 40% disability, were included in the study. There was incomplete information about nine respondents who were orphans and mentally disabled. Therefore, the number of respondents included in the study results was 261. Among 28 study subjects, respondents were mothers/interpreters/teachers. In the rest of the cases, WWD themselves answered the questions. For IDI, those respondents were selected, who consented to the same and were vocal about it. The principle of redundancy was followed to obtain the desired sample size. Data collection was continued till no new information emerged. For qualitative analysis, themes were drawn from the verbatim told by respondents. After reading and rereading the data, preliminary ideas were noted. Data were manually coded by separating words and sentences verbatim. By identifying common patterns, codes were collated into possible themes. All the data was put together related to each possible theme.

Ethical clearance was taken from the Institute Ethics Committee (Intramural), Post-Graduate Institute of Medical Education and Research, Chandigarh, India vide its reference number- Histo./14/884 dated 24/3/14. Permission from the hospital authorities was also taken. WWD were explained the purpose of the study. The identity of the participants was kept secret. Participants were asked to sign the consent sheet/mark their thumb impression (in the case of illiterate or visually impaired respondents). In the case of blind/mentally disabled/mentally ill respondents, information was sought from the mother/caregiver or close relative of respondents whose signatures were taken on the assent form.

## Results

Lack of access was the main barrier reported by WWD to OBG care in hospitals. Most hospitals lacked adequate provisions for PWD. Proper ramps, stairs, toilets, etc. were not present as denoted by the verbatim responses. Table 2 shows that hospital 5 (H5) had the highest score (94.3). Even this score falls in the average grade. Other hospitals scored poorly in terms of the accessibility of OBG services for WWD. All the hospitals scored low on accessibility services.

**Table 2:** Study domains and their sub-domains with accessibility score of obstetrics and gynecology (OBG) services in selected hospitals * H1-H5= Hospital number 1-5

Study domains and sub-domains (Maximum attainable score)	The score obtained by hospitals (H1-H5)*				
	H1	H2	H3	H4	H5
Evaluation of hospital services (40)	4.3	2.3	2.8	3.3	4.3
Information and communication (15)	0.3	0.3	0.8	2.3	0.3
Accountability (17)	1	1	1	1	1
Special arrangements (8)	3	1	1	0	3
External environment (41)	4	5	5	0	13
Parking (14)	1	1	1	0	1
Taxi stand (2)	0	0	0	0	0
Alighting (2)	0	0	0	0	1
Pathways (14)	3	4	4	0	10
Curb cuts (4)	0	0	0	0	0
A pedestrian crossing (2)	0	0	0	0	0
General obstructions (3)	0	0	0	0	1
Internal environment (199)	56	63.5	62	37	77
Main entrance (10)	4	6	4	5	6
Doors (19)	8	5	3	6	4
Corridors (12)	3	4	3	1	8
Elevators (20)	10	10	14	0	13
Steps/ stairs (18)	7	9	12	8	8
Ramps (13)	7	7	9	7	11
Handrails(10)	0	0	5	0	5
Escalators/passenger conveyors (9)	0	0	0	0	0
Toilets (15)	1	7.5	0	0	0
Drinking water (7)	1	1	1	1	3
Eating outlets/cafeteria (11)	2	0	0	0	3
Public telephones (3)	0	0	0	0	0
Resting facilities (8)	1	3	2	2	4
Reception and information counters (14)	5	4	3	3	4
Signage (7)	3	3	6	4	5
Approach to shops/chemists (6)	1	0	0	0	3
Approach to the bank (4)	2	4	0	0	0
Emergency evacuation (13)	1	0	0	0	0
Overall accessibility score of obstetrics and gynecology (OBG) services in selected hospitals (280)	64.3	70.8	69.8	40.3	94.3

External environment

Physical barriers often led to the loss of compliance among WWD; the husband of a respondent lamented, “It is very difficult to bring her to hospital. She gets on the tempo with great difficulty”. A 60-year-old married respondent told, “I am not able to get into a bus. I face difficulty in boarding it. I wish there were steps or ramp on the bus”. Another one said, “It is difficult to go alone. Right from home to the hospital, it seems difficult. At least, there should be some transport”. There was no provision for special transport for WWD. They had to arrange their vehicle, adding to the cost of treatment, as one respondent said, “ We came from our hometown in a special auto-rickshaw. I cannot come on the bus due to my pregnancy".

Parking for the general public was far off from the hospital building making it difficult for PWD to negotiate the gap. As one of the respondents reported, “We face difficulty in parking. It is far away from the hospital department. It is a problem to come to the hospital”.

Internal environment

Another respondent highlighted multiple problems like lack of proper toilets, equipment, and assistance: “I bring my own toilet seat; the wheelchair pinches. I have to be lifted to be put on the ultrasound machine. No hospital attendant helps while I am being examined. My relatives come with me”. Respondents faced trouble climbing stairs. In most hospitals stairs had no accessibility features as denoted by verbatim responses of WWD: “In the hospital too, it is difficult to go up the stairs”; “It would have been better if there was an escalator, lift or ramp in the department”. Another respondent stated that she had a problem walking and was fed up with coming to the hospital for treatment, “I have to walk a lot, sitting on the feet. It is very difficult. I am fed up with this treatment”. Even if wheelchairs were available, WWD reported waiting for hours to get wheelchairs/stretchers as denoted by their verbatim, “We waited for 1/2 hour to get a wheelchair. It was not available”. Another respondent told, “Here, in the hospital, my mother stands in the queue. Inside too, the turn comes based on the card number allotted. A full day is wasted when we come here”.

There was no special equipment in the OBG department to examine WWD. Patients had to struggle to get onto examination tables. They had to be lifted manually up to the table by their family members as reported by the husband of a respondent, “It is difficult to take her alone for Ultrasound. Hence, we did not go again. She has to be physically held all the time”. A 35-year-old married respondent with a locomotor disability said, “The ultrasound machine in the hospital is too high. So, I have to call my husband. The staff objects to this”.

Overcrowding of hospitals was another barrier reported by some (11.5%) WWD. There were no special lifts for PWD and other lifts generally remained overcrowded, as one respondent said, “I feel suffocated in the elevator due to overcrowding so I prefer to take the stairs. It is difficult to stand for long in overcrowded hospitals”.

Lack of empathy for PWD was highlighted by a respondent, “No doubt it is written on the notice board that after every four patients, staff or disabled people should come; but this practice is not seen. Staff never ends”. Unfriendly toilets were reported as a barrier by a few (5.75%) of the respondents: “Toilets are not clean. Special toilets should be there". Often the barriers in hospitals were accepted by them as their fate; they seemed to have learned to manage all by themselves and not to report the issue to anyone: “There is no other way. We will have to cope with our problems".

## Discussion

Of late, disability as a human rights issue has moved from a marginal to mainstream agenda. Access to optimum health care services is vital for every woman. WWD face multiple discrimination and social exclusion on this issue. They face health issues like any other woman. Previous studies suggest that due to various environmental barriers, negative attitudes of people, and fewer social opportunities, WWD become socially disconnected and isolated from various spheres of life [4]. A study in Ludhiana, India highlighted that the public transport system was least accessible for PWD [5,6]. There were no special help desks for WWD in the study hospitals. They had to search for various services on their own. The external environment was also not friendly to PWD. There was no proper parking, pathways, or alighting point for PWD visiting hospitals. Every access point was full of obstructions making it difficult for WWD to use the health facility. The approach to the hospital entrance was also full of barriers. Elevators remained overcrowded with patients. There was no special arrangement for PWD. Stairs and ramps were not following the accessibility standards. There were no separate toilets for WWD. Access to the drinking water facilities was also obstructed. There were counters reserved for PWD but their height was not as per the recommended standards. It seems that a ‘lip service’ was done for ‘appearance-sake’ to make the facilities look PWD-friendly. No sincere intention to facilitate access to PWD was visible in any hospital. There was no proper signage for PWD in any hospital. OBG services were not responsive to the needs of WWD. Overall, one would gather the impression that PWD was not welcome in hospitals.

Previous studies have also indicated that the physical environment is largely inaccessible for WWD in India [7-9]. A previous study also reported that despite continued lobbying, accessibility in private and public buildings in India is limited due to a lack of funds or prioritization. In the US also, reasons for inadequate health care services for PWD included transportation problems, financial barriers, inaccessible buildings, lack of knowledge, and bad attitude of health care providers [10]. In Sierra Leone, people with severe disabilities had less access to public health care services than non-disabled people [11]. In our study, lack of access to WWD to OBG care in hospitals was the main barrier reported by the respondents. Hospitals lacked any special provisions for PWD, e.g., ramps, stairs, toilets, etc. The transport system was not friendly to WWD, who depended upon others for assistance. In Malaysia, none of the hospitals satisfied 100% accessibility criteria for PWD. Parking was not friendly for PWD. Toilet facilities were not amenable to independent use by PWD. Private hospitals scored better than public ones [12]. The health of WWD is unnecessarily compromised by an unfriendly transport system, lack of access to services, inaccessible equipment, and lack of training among health care providers [13-20]. WWD were often carried wrapped in a bed sheet to move between areas due to the non-availability of wheelchairs [17]. WWD reported access barriers like high examination tables during physical examinations [21-23]. Our respondents expressed the need of creating enabling environment by modifications in the present hospitals to make them disabled-friendly supplemented by the training of health care providers to deal with such patients. They suggested that physical accessibility should be improved in hospitals to make them barrier-free. Physical barriers to accessing hospital services reflect a simple lack of awareness and the assumption that “it costs too much” to remove these barriers. Changing misperceptions and prejudiced attitudes are more difficult to address than removing physical barriers [23].

Limitations of the study

This study was based in an urban area. It is likely that the barriers faced by urban-based WWD might be dissimilar from those experienced by WWD residing in rural areas. In fact, the inaccessibility of health facilities could be more marked for PWD in rural regions. It is likely that the barriers faced by urban-based WWD might be dissimilar from those experienced by WWD residing in rural areas, an area that requires further study. 

Strength of the study

This is the first study on the subject from India.

## Conclusions

Hospitals are part of general society. Barriers faced in hospitals reflect obstacles in society also. In this study from north India, hospitals scored low in physical accessibility and friendliness towards WWD. Hospitals lacked any special provisions for PWD. Inaccessible hospitals result in the exclusion of PWD from their ambit. An accessible healthcare system without obstacles is a prerequisite to mainstream WWD. The provision of facilities to WWD pertaining to education, work, and freedom of mobility is useless without ensuring healthcare services that are accessible to them. As the number of WWD would increase due to increasing lifespan, it is essential to ensure optimum SRH for this group.
